# Utilization, Contributions, and Perceptions of Paid Home Care Workers Among Households in New York State

**DOI:** 10.1093/geroni/igac001

**Published:** 2022-01-06

**Authors:** Madeline R Sterling, Joanna Bryan Ringel, Jacklyn Cho, Catherine A Riffin, Ariel C Avgar

**Affiliations:** 1 Division of General Internal Medicine, Department of Medicine, Weill Cornell Medicine, New York, New York, USA; 2 Division of Geriatrics and Palliative Medicine, Department of Medicine, Weill Cornell Medicine, New York, New York, USA; 3 College of Industrial Labor Relations, Cornell University, Ithaca, New York, USA

**Keywords:** Home care worker, Home health aides, Home health care, Quality of home care

## Abstract

**Background and Objectives:**

While family caregivers have traditionally provided care for older adults with chronic conditions and disabilities, the demand for paid home care workers has increased in the last decade. Although typically thought to assist with personal care, emerging data suggest that paid home care workers assist with a wider scope of care. However, the extent and quality of the care they deliver remains poorly understood.

**Research Design and Methods:**

Using the Empire State Poll, a telephone-based cross-sectional survey of 800 adults in New York State, we characterized the types of care that paid home care workers provided and the perceived value of that care.

**Results:**

Of 800 participants surveyed, 274 reported that they or an immediate family member received care from a paid home care worker (34.3%). Of these, the majority (73.9%) reported that paid home care workers provided emotional and/or medical care, in addition to personal care. In adjusted models, providing emotional and medical care (compared to personal care alone) was associated with nearly a twofold greater perception of importance and experience by the care recipients.

**Discussion and Implications:**

Our findings provide additional data on how paid home care workers contribute to patient care, from the perspective of the care recipient(s). The type of care provided is associated with varying magnitudes of perceived quality. Although limited to New York, these findings have implications for paid home care workers’ training and compensation. Future studies are warranted to investigate the specific factors that mediate the association between types of care provided and their perceived value.

**Translational Significance:** Although thought to provide mostly personal care, paid home care workers were also found to assist community-dwelling adults with medically oriented care and provide emotional support. Providing these additional types of care was associated with greater perceptions of quality of care by care recipients.

Older adults as well as those with chronic conditions and disabilities commonly rely on caregivers for help at home with their personal and medical care. While family caregivers have traditionally provided the backbone of this care, over the last decade, the demand for paid home care workers has increased substantially (1–3). There are currently 2.3 million paid home care workers, and the field is projected to grow by more than 1.5 million by 2030 (2). Paid home care workers, which include home health aides and attendants as well as personal care aides, provide personal assistance and health care support to older adults and people with chronic conditions and disabilities in their homes (2, 4).

Although typically thought to provide only personal care, emerging data suggest that paid home care workers assist with a wider scope of care activities beyond activities of daily living and instrumental activities of daily living. Specifically, qualitative studies have found that paid home care workers, especially home health aides, often assist with medical care, such as the maintenance and management of chronic conditions and acute complaints (5–7). Additionally, patients and family caregivers have reported that paid home care workers provide emotional support and companionship to them or their loved ones (8, 9). Despite these contributions, few studies have quantitatively documented the types of care that paid home care workers provide on a day-to-day basis and the association between the types of care they provide and its quality. A better understanding of how they contribute to patient care and their perceived value is important because this workforce is increasingly caring for medically complex adults who are choosing to age in place, and because payment models in home care are shifting toward reimbursement based on quality metrics. If paid home care workers are indeed providing more medical or emotional care than previously thought, increased awareness of their contributions by the medical community and policymakers is needed, and formalized training will need to appropriately reflect these contributions. Furthermore, evidence supporting these expanded roles has clear implications for paid home care workers’ professional development and payment. Studies have consistently found that paid home care workers feel marginalized, unappreciated, undervalued, and poorly integrated into the health care team (10–12). Without data that speak to the value of their contributions to patient care, these issues are unlikely to change.

To that end, the objectives of this study were threefold: (a) to determine the utilization of paid caregiving among households in New York State, (b) to determine the types of caregiving activities in which paid home care workers contributed, from the perspective of the household, and (c) to examine the association between the types of care provided by paid home care workers and the value of that care, as measured by its importance and overall experience from the perspective of the care recipient(s).

## Research Design and Methods

### Study Design

We conducted a cross-sectional study analyzing data collected as part of Cornell Survey Research Institute’s 2020 Empire State Poll (13). Institutional review board approval was obtained in January 2020 by the Survey Research Institute (SRI) at Cornell University.

### Survey Development

The Empire State Poll is an annual telephone survey of randomly selected adults aged 18 and older residing in New York State. Each year the survey includes a core set of questions about community, government, and economic issues as well as demographic data. In addition to the core questions, investigators may submit questions to be included in the annual survey. Submitted questions are reviewed by the SRI and pilot-tested with a sample of 25 individuals who do not participate in the final survey. Investigators are given feedback regarding any issues encountered during pilot testing and offered the opportunity to modify their questions for clarity and comprehensibility prior to launch of the final survey, which our team did. In 2020, the final survey contained 62 interview questions, including 13 submitted by this research team.

### Survey Administration

The Empire State Poll was administered from January 24, 2020 to March 15, 2020. A total of 18 007 phone records were contacted to achieve the final sample size of 800 individuals (unique households; Supplementary Material I). The 2020 Empire State Poll had a cooperation rate (defined as the number of completed surveys divided by the number of potential interviews) of 25.6% and a response rate (defined as the number of completed surveys divided by the total eligible sample) of 14.6%. The average interview length was 18 min. The Cornell SRI used a dual-frame random digit dial sampling of landlines and cell phones in New York State. An additional sampling frame was added to recruit individuals with out-of-state cell phone numbers. Additional oversampling was utilized to ensure representation of Black and Hispanic individuals throughout New York State, as well as upstate and downstate geographic regions sampling was conducted in proportion to population totals.

### Data Collection

Research assistants used a standard Computer-Assisted Telephoning Interviewing software package called CASES to call prospective participants. This software was used to collect and store sample information and measure call outcomes. All data were de-identified by removing confidential information and stored in a private and secure database on SRI’s network, monitored by the Cornell Information Technology team.

### Survey Questions About Paid Caregiving

Informed by The Convoy of Care Model, which theorizes the intersection of unpaid and paid caregiving and its impact on caregiver and care recipient outcomes, we developed a total of 13 questions that assessed public utilization of and attitudes toward unpaid and paid caregiving for personal and health-related needs; of those, 8 questions are relevant to this study (14). We asked all participants: (1) *Have you or an immediate family member ever had a paid home care worker?* A “yes” response triggered 7 additional questions that began with: *Our next few questions are about your most recent experience with a paid home care worker*: (2) *Approximately how many hours per week did the paid home care worker spend with you or your family member?* (3) *How did you or your family member pay for the home care worker? (insurance through Medicare, insurance through Medicaid, privately without insurance, and other)*. (4) *Which of the following types of activities did the paid home care worker assist with? (personal care [such as assistance with bathing, dressing, meal preparation, light housekeeping, and shopping], medical care, emotional support, and/or other)*. *For this question, participants could select as many of these prespecified types of activities that applied to their experience with a paid worker. These categories were derived from the existing literature on paid home care workers’ contributions to care.* Informed by frameworks on the benefits and value of receiving care (15, 16), we also asked participants: (5) *How important was the home care worker to the overall care that you or your family member received? (not at all important, slightly importantly, somewhat important, very important)*. (6) *How would you rate the overall experience of having a paid home care worker? (very negative, somewhat negative, neither positive nor negative, somewhat positive, very positive)*. (7) *How connected, if at all, was the home care worker to the person they cared for? (not at all connected, slightly connected, somewhat connected, very connected)*. (8) *Would you consider using a paid home care worker again if needed? (yes, no, not sure)*.

### Main Exposure

The types of caregiving activities in which paid home care workers were engaged (personal care, medical care, emotional support, other), as assessed with the 1-item survey question above (#4).

### Main Outcome

The main outcome of the study was the value that paid home care workers’ care provided. We measured value with 2 separate constructs, one pertaining to (a) the importance of having a paid home care worker (as measured by the 1-item survey question above, #5) and a second pertaining to (b) the overall experience of having a paid home care worker (as measured by the 1-item survey question above, #6).

### Data Analysis

We assessed the distribution of demographic and caregiving characteristics among participants who reported that they or their family members received care from a paid home care worker. We next assessed differences in demographic and caregiving characteristics across the types of care that the paid home care worker provided (personal, emotional, medical) using chi-square, Fisher’s exact, and analysis of variance tests. We used robust Poisson regression to analyze the association between type of care provided and importance of the paid home care worker, adjusting for demographic/caregiving characteristics. We used Poisson regression because with frequent outcomes, prevalence ratios (PRs) are less likely to be overestimated and are easier to interpret than odds ratios (17). Characteristics were selected for the model based on previous literature (7) or a significant bivariate association (*p* < .05) with type of care. We repeated this process for the remaining outcome of experience with a paid home care worker.

Covariates with missing data included method of payment for paid care (5%), rural residence (5%), hour of paid care (4%), and race (3%). Sample weights were applied to account for the sampling design and permit generalization to the entire state. The sample size of 800 produces a margin of error of ±3.5 percentage points. Data management and modeling were conducted using STATA version 16 (StataCorp LLC, College Station, TX).

## Results

Of the 800 ESP participants, 274 reported that they or an immediate family member (household) had received care from a paid home care worker in the past (34.3%). For the main analyses, and given the study objectives, we excluded participants (*n* = 6) who did not provide information on the types of care that the paid home care worker provided, and 29 participants who reported that the paid caregiver did not assist with personal care. A total of 239 participants comprised the final analytic cohort (Supplementary Material II).

### Characteristics of the Sample

Characteristics of the 239 participants who reported that they or their family member received care from an HHW are given in Table 1. They had a mean age of 48.6 (95% confidence interval [CI]: 45.8–51.3), 58.7% were female, 73.4% were White, 15.6% were Black, 33% had income less than $50 000, and 86.5% lived in urban areas of New York. With respect to paying for the paid home care worker, nearly one quarter (23.8%) reported using Medicare insurance, 18.8% Medicaid, and 40.6% reported paying privately without insurance. They reported receiving paid care for a mean of 34.3 (95% CI: 28.5–40.1) hours a week and 16% and 46.5% reported that the paid home care worker was “somewhat” and “very” connected to the person they cared for, respectively. With respect to the importance of the paid home care worker to overall care received, nearly three quarters of the participants (74.1%) reported that the HHW was “very important.” When asked about the overall experience of having a paid home care worker, 31.1% and 54.5% reported that the experience was “somewhat” and “very” positive, respectively.

**Table 1.** Characteristics of Study Participants*

Characteristic	*N* (%)	Mean (95% CI)
*N*	239	
Age of participant		48.6 (45.8–51.3)
Number of adults 65+ in household		0.5 (0.4–0.6)
Female gender	129 (58.7)	
Race		
White	178 (73.4)	
Black	31 (15.6)	
Asian	5 (3.5)	
Other	20 (7.5)	
Rural residence	23 (13.5)	
Geographic residence		
Urban	205 (86.5)	
Large rural	12 (2.6)	
Small rural	6 (8.7)	
Isolated	5 (2.2)	
Hispanic	31 (14.8)	
Income <$50 000	62 (33)	
Less than high school education	8 (5.3)	
Married	113 (49.2)	
Number of children in household		0.6 (0.3–0.8)
Foreign born status		
Born in the United States (or a territory)	211 (85.5)	
Born abroad to at least one American parent	7 (5.2)	
Neither born in the United States (nor a territory) nor to an American parent	21 (9.4)	
Employment status		
Full-time, all year round	125 (52)	
Part-time	32 (16.5)	
Retired	45 (13.4)	
Disabled or unable to work	10 (7.1)	
Not working	26 (11)	
Hours worked last week		26.8 (22.7–30.9)
Method of payment for paid care		
Insurance through Medicare	56 (23.8)	
Insurance through Medicaid	34 (18.8)	
Privately paid, without insurance	102 (40.6)	
Other	36 (16.9)	
Hours of paid care/week		34.3 (28.5–40.1)
How connected was home care worker to the person cared for?		
Not at all connected	4 (1.1)	
Slightly connected	14 (12.1)	
Somewhat connected	28 (12.7)	
Very connected	193 (74.1)	
Important of home care worker to the overall care?		
Not at all important	26 (15)	
Slightly important	38 (22.6)	
Somewhat important	58 (16)	
Very important	115 (46.5)	
Overall experience of having a paid home care worker		
Very negative	5 (2.5)	
Somewhat negative	7 (3.5)	
Neither positive nor negative	19 (8.5)	
Somewhat positive	77 (31.1)	
Very positive	131 (54.5)	

*Note:* CI = confidence interval.

*Participant who responded to the survey may or may not be the one receiving care.

### Types of Care Performed by Paid Home Care Workers

**Figure 1.**
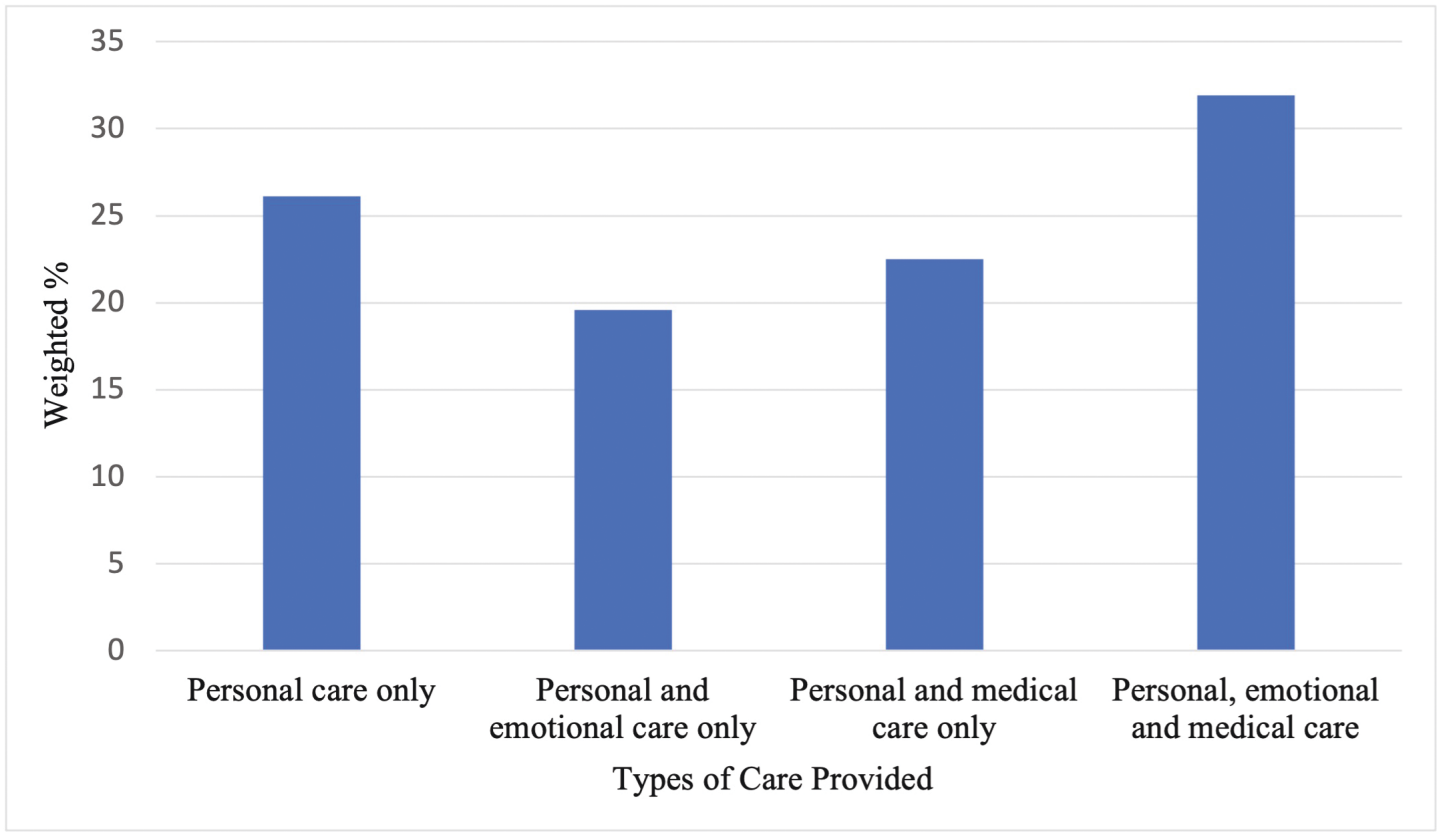
Types of care provided by paid home care workers.

Of the 239 participants, 26.1% reported that the paid home care worker assisted with personal care only, 19.6% reported that the paid home care worker assisted with personal and emotional care only, 22.5% reported that the paid home care worker assisted with personal and medical care only, and 31.9% reported that the paid home care worker assisted with personal, emotional, and medical care (Figure 1).

How care was paid for differed significantly by the type of care rendered; for example, personal care only was more likely to be paid for privately (without insurance), whereas personal and medical care only were more likely to be paid for by Medicare (Supplementary Material III). Additionally, the number of hours of care differed significantly by the type of care rendered; participants who reported receiving personal, emotional, and medical care had more hours of paid help on average (46.9 hours/week), compared to those who received personal care only (25.4 hours/week), personal care and emotional care only (36.8 hours/week), and personal and medical care only (24.2 hours/week), *p* < .04 (Table 1).

### Importance of Paid Home Care Worker to the Overall Care Received

In unadjusted models, paid home care workers who performed other types of care (emotional and/or medical) beyond personal care alone were associated with greater perceptions of importance (Table 2). That is, participants with paid home care workers who provided emotional and personal care were 80% more likely to report that the paid home care worker was “very important” compared to paid home care workers who performed personal care only (PR 1.80 [95% CI 1.00–3.28]). Similarly, paid home care workers who provided personal, emotional, and medical care were 2 times more likely to be viewed as “very important” compared to paid home care workers who provided only personal care (PR: 2.09 [CI: 1.18–3.72]). These associations remained significant, albeit of slightly less magnitude, after covariate adjustment including demographics of the participant and household, as well as the number of hours in which care was provided and payment method for care.

**Table 2.** Association Between Tasks Performed by Paid Home Care Worker and the Perception of Paid Care as Very Important

Tasks	Crude		Model 1	
	PR	*p*	PR	*p*
Personal care only	Ref		Ref	
Personal and emotional care only	1.81 (1–3.28)	.051	1.64 (1.05–2.58)	.03
Personal and medical care only	1.47 (0.77–2.82)	.24	1.66 (1.05–2.62)	.03
Personal, emotional, and medical care	2.09 (1.18–3.72)	.01	1.85 (1.21–2.83)	.00

*Notes:* PR = prevalence ratio. Crude Model: unadjusted. Model 1: adjusts for age of respondent, number of adults aged 65 and older in household, female, race, rural residence, Hispanic, married, method of payment for paid care, and hours of paid care/week.

### Experience of Having a Paid Home Care Worker

The association between the care performed by the paid home care worker and how positive the experience of having a paid home care worker was varied by the type of care the paid home care worker performed (Table 3). Having a paid home care worker who provided both personal and emotional care was associated with an 80% greater likelihood of reporting a positive experience by participants compared to having one who provided emotional care personal care only (PR: 1.80 [CI: 0.82–3.94]). Similarly, having a paid home care worker who provided personal, emotional, and medical care was associated with a 94% greater likelihood of reporting a positive experience than having one who provided personal care only (PR: 1.94 [CI: 0.90–4.18]). Notably, having a paid home care worker who provided personal and medical care only (and not emotional care) was associated with 38% more positive experience than having one who provided personal care only (PR: 1.38 [CI: 0.57–3.33]); however, this association did not achieve statistical significance. Likewise, in adjusted models, the association between tasks performed by a paid home care worker and having a very positive experience remained positive and significant when emotional care was rendered in addition to either personal care or in addition to personal and medical care; adjusted models accounted for the demographics of the participant and household, as well as the number of hours in which care was provided and payment method for care.

**Table 3.** Association Between Tasks Performed by Paid Home Care Worker and the Perception of Paid Care as a Very Positive Experience

Tasks	Crude		Model 1	
	PR	*p*	PR	*p*
Personal care only	Ref		Ref	
Personal and emotional care only	1.8 (0.82–3.94)	.051	1.96 (1.18–3.26)	.03
Personal and medical care only	1.38 (0.57–3.33)	.24	1.35 (0.75–2.44)	.03
Personal, emotional, and medical care	1.94 (0.9–4.18)	.01	1.88 (1.16–3.05)	.00

*Notes:* PR = prevalence ratio. Crude Model: unadjusted. Model 1: adjusts for age of respondent, number of adults aged 65 and older in household, female, race, rural residence, Hispanic, married, method of payment for paid care, and hours of paid care/week.

## Discussion

In this cross-sectional and representative survey of community-dwelling adults in New York State, a third of participants reported that their household received care from a paid home care worker. Although a quarter of participants reported that the worker provided personal care alone, the majority of participants reported that paid home care workers provided emotional and/or medical care, in addition to personal care, to them or their immediate family member. In general, satisfaction with the care provided by paid home care workers was high, as was the overall experience of having a paid home care worker. In multivariable models, we found that the type(s) of care provided by the paid home care worker was associated with different perceptions of value by the care recipient. That is, when more than personal care was provided (eg, emotional and/or medical), we found associations with greater perceptions of importance and a positive experience by the care recipient. Notably, providing emotional care (in addition to personal care) increased the magnitude of these associations more than the provision of medical care without emotional care.

This study adds to the existing literature in 2 main ways. First, it improves our understanding of paid caregiving utilization and how specifically paid home care workers contribute to patient care, from the perspective of the care recipient(s). Previously, paid home care workers’ contributions were inferred based on their job title and the formal scope of care and training requirements associated with their job title (18, 19). This, however, is not always an accurate depiction of the care they provide, as demonstrated by a recent qualitative study by Reckrey et al. (5) of patient–paid caregiver dyads. This study found that in the course of their day-to-day activities, agency-employed workers went well beyond assisting patients with personal care, often supporting patients with the management of their chronic conditions, promoting their general health, and fostering their mental health. Other qualitative studies have found the same, including a study of family caregivers by Shaw et al. (20) which found that paid home care workers often provided patients with functional and emotional support. Similarly, a recent survey by Sterling et al. (7) of over 300 paid home care workers found that nearly two thirds contribute to their patients’ heart failure self-care activities, such as assisting with the preparation of low salt meals, reminding patients to take medication, and engage in physical activity. Our study expands these qualitative findings by quantitatively documenting the contributions of paid home care workers from the care recipients’ perspective. Future studies are needed to determine if these findings persist nationally, because the scope of care of paid caregivers often varies by state.

A second way in which our study adds to the literature is that it is one of the first to demonstrate that the type of care paid home care workers provide is associated with varying magnitudes of perceived value or quality. This is important because, despite anecdotal observations of this association, quantitative data have been lacking (21). Not only do our findings highlight the value of paid home care workers’ contributions to patient care, but they are also important from a payment and policy perspective, both at the individual and organizational levels. Under the revised Fair Labor Standards Act, agency-employed workers are paid at least the federal or state minimum wage, and this pay is based on time, not on care contributions (22). Currently, 24% of paid home care workers live in households below the federal poverty line, compared to 9% of all US workers (2). Thus, to the extent that this workforce is providing care that is not being captured, acknowledged, or recognized, there is a need to reassess compensation models that will account for this contribution to patient care. Related, but separate to this, is that payment for Medicaid and Medicare-funded home care are increasingly shifting away from traditional fee-for-service models to value-based payment (VBP). VBP, which aims to contain health care costs while improving quality, is designed to reward home care agencies for the quality of services rendered to patients, rather than the volume of those services (23). Existing quality metrics for home care, however, are mostly process measures (eg, vaccination given) and utilization outcomes (eg, hospitalization; improvement in activities of daily living function) (24). Although the “overall quality” of home care is assessed, it is not specific to the contributions of the home care workers themselves (eg, the types of care provided or intensity of that care), suggesting that payment models may not sufficiently measure the quality of home care services (25). Our findings suggest that more comprehensive metrics may be needed to inform payment models for agency-employed home care workers.

Notably, we found that providing emotional support was associated with greater perceptions of value. This adds to existing frameworks from the family caregiving literature, whereby the provision of emotional support is associated with better perceptions of care and higher-value care by care recipients (15, 16). Research has demonstrated that family (unpaid) caregivers provide psychosocial support, including emotional and mental support (eg, touching, listening, attention, humor, assist with referral to mental health services), social and spiritual support (eg, respect spiritual needs, empathy, assist with attending religious or spiritual services), and coping with symptoms of disease (eg, anxiety, depression) (26, 27). Parallel to this, studies have shown that paid home care workers have also participated in these activities for adults during coronavirus disease 2019 (COVID-19) (12), as well as for adults with high-need medical conditions, such as dementia, end of life, human immunodeficiency virus/acquired immunodeficiency syndrome, and heart failure (6, 8, 28). However, the specific components of the emotional-related care that paid home care workers provide and how that influences the care recipients’ perceptions of care remain unknown from this study. Additionally, it is unclear what, if any, training, these workers receive regarding emotional care and the toll that providing this care takes on their own health and well-being. For example, if paid home care workers are helping patients combat depression or anxiety in the home, then they may require additional training on this topic and support for the consequences that this type of caregiving has on their own work experiences. This is especially relevant during the COVID-19 era, where the prevalence of mental health problems and social isolation is increasing among adults receiving unpaid and paid care (12).

## Implications

Our findings have implications for the workforce, policies that govern payment and training, and future research. Despite providing day-to-day hands-on care, paid home care workers have historically felt invisible to the medical field and society at large (29). As demonstrated here, and from the perspective of their clients, these workers make substantive contributions beyond the personal care that they are often perceived to provide. Our findings raise awareness of the contributions of this workforce, and further, by demonstrating how these contributions are associated with value, they can inform policies to realign reimbursement for paid home care workers’ wages and services (30). For example, state-level policies that govern home care curriculum and training may need to be adapted to reflect their current contributions, particularly with respect to emotional and certain medical aspects of care (31), which they may not receive standardized training on currently. Our findings also have implications for federal-level policymaking, for example, the Biden administration’s recent American Jobs Plan, which includes an estimated 400 billion dollars of investments in home care workers over an 8-year period. This proposal seeks to expand home care in the United States while increasing the quality jobs for paid home care workers. Our findings provide a framework for investments in the expanded role that these caregivers play in a manner that would enhance quality of care and working conditions for this essential workforce, by highlighting 3 essential and interrelated categories of care (32). Finally, the mechanism underlying the associations between the types of assistance provided and the perceived quality of that care warrants further investigation. Future studies might investigate what factors explain or mediate this association, such as individual-level characteristics (eg, sociodemographic characteristics, experience level, attitude, and training of the paid home care worker and the clinical need and severity of the care recipient), interpersonal characteristics between the paid home care worker and the care recipient (eg, connectedness, trust, cultural fit), and factors pertaining to the employment of the worker (eg, method of payment for paid care, duration of services, and agency vs nonagency employment).

### Limitations

Our study has limitations that warrant consideration. First, although we asked participants to report on their most recent experiences with a paid home care worker, we lack specific data on the temporality of the relationship, the duration in which the worker provided services and for whom (in the family unit), and specificity regarding the types of care within broad domains (eg, emotional support) that were provided. Second, the Empire State Poll did not include measures of participants’ functional and clinical status, which are important determinants of the types of care provided by paid caregivers. Third, we lacked data on the personal characteristics of the paid home care workers, the relationship between them and their care recipients (eg, connectedness, cultural fit, trust, and objective payer data for the services they provided; these factors are likely to influence the degree to which households were satisfied with paid home care workers, but we were unable to account for these in our models. Future studies would benefit from including these additional data. Finally, although a representative random sample, participants were residents of New York State and the response rate was modest, thus their experiences may not be wholly generalizable.

## Conclusion

In this cross-sectional survey of New York State households, we found that one third of households utilized a paid home care worker. Paid home care workers often provided care beyond personal care alone, including emotional and/or medical care. Doing so was associated with greater perceptions of importance and positive experiences, by the household. Future studies are needed to understand whether these findings hold at the national level, and programs and policies that better match their contributions in the home to their training and payment are warranted.

## Supplementary Material

igac001_suppl_Supplementary_Material
