# Supplementary material for: Utilization, Contributions, and Perceptions of Paid Home Care Workers Among Households in New York State
Source: Innov Aging. 2022 Jan 6;6(2):igac001. doi: 10.1093/geroni/igac001 (PMC8883505; doi:10.1093/geroni/igac001)
Supplement: igac001_suppl_Supplementary_Material [file igac001_suppl_supplementary_material.docx]

***Innovation in Aging* Online Supplementary Material: Sterling, M. R., Ringel, J. B., Cho, J., Riffin, C. A., & Avgar, A. C. Utilization, Contributions, and Perceptions of Paid Home Care Workers among Households in New York State.**

**Supplemental Material I. Final Sample Status for the 2020 Empire State Poll**

| **Status** | **Downstate** | **Upstate** | **Total** |
| --- | --- | --- | --- |
| Completed Survey | 400 | 400 | 800 |
| Refusal | 1439 | 868 | 2307 |
| Non-contact | 478 | 710 | 1188 |
| Partial | 14 | 8 | 22 |
| Incapable | 5 | 8 | 13 |
| Language Problem | 92 | 8 | 100 |
| Unknown Eligibility | 2161 | 1602 | 3763 |
| Not a NYS Resident | 300 | 192 | 492 |
| Nonworking Number | 4094 | 4731 | 8825 |
| Non-Residence | 174 | 260 | 434 |
| Age Ineligible (<18) | 34 | 19 | 53 |
| Ineligible – Region quota | 0 | 10 | 10 |
| Total Sample Used | 9191 | 8816 | 18007 |
| Response Rate^2^ | 13.1% | 16.4% | 14.6% |
| Cooperation Rate^3^ | 21.6% | 31.3% | 25.6% |

^2^ AAPOR Standard Definitions. Final Dispositions of Case Codes and Outcome Rates for Surveys. (http://www.aapor.org/AAPOR_Main/media/publications/StandardDefinitions20169theditionfinal.pdf)

^3^American Association for Public Opinion Research (AAPOR) response rate and cooperation rate calculations. The response rate is the total number of survey completions divided by the total eligible sample (total sample minus all ineligible, non-households, and estimated proportion of households where eligibility was not determined). Cooperation rate is the total number of survey completions divided by the number of potential interviews (this includes all instances where contact was made with a properly selected person, but not including those instances where the respondent was incapable of cooperating due to language or physical limitations).

**Supplemental** **Material II. Exclusion Cascade**

n=274

ESP Sample

N=800

n= 268

Participant answered “no” to the question “Have you or an immediate family member ever had a paid home care worker?” (n=526)

Missing information on questions regarding tasks performed by home care worker (n=6) or importance of home care worker (n= 0)

Participant reported that home care worker did not perform personal tasks (n= 29)

n= 239

| **Supplemental Material III. Characteristics of Study Participants by the Type(s) of Care Provided by the Paid Home Care Worker** | | | | | |
| --- | --- | --- | --- | --- | --- |
| Characteristics | Personal care only | Personal and emotional care only | Personal and medical care only | Personal, emotional, and medical care | p-value |
| N | 47 (26.1) | 59 (19.6) | 46 (22.5) | 87 (31.9) |  |
| Method of payment for paid care |  |  |  |  | 0.04 |
| Insurance through Medicare | 11 (14) | 15 (26.7) | 15 (35.8) | 15 (23.2) |  |
| Insurance through Medicaid | 4 (20.4) | 8 (8.2) | 7 (19.5) | 15 (24) |  |
| Privately paid, without insurance | 23 (56.5) | 29 (46.1) | 10 (9.2) | 40 (41.5) |  |
| Other | 7 (9) | 7 (19) | 9 (35.4) | 13 (11.3) |  |
| Hours of paid care/week, mean (SD) | 25.4 | 36.8 | 24.2 | 46.9 | 0.00 |
